# Nonossifying fibromas of the distal tibia: possible etiologic relationship to the interosseous membrane

**DOI:** 10.1007/s11832-016-0745-5

**Published:** 2016-06-03

**Authors:** David A. Muzykewicz, Amanda Goldin, Nicholas Lopreiato, Katie Fields, John Munch, Jerry Dwek, Scott J. Mubarak

**Affiliations:** Department of Orthopedic Surgery, Rady Children’s Hospital, 3030 Children’s Way, Suite 410, San Diego, CA 92123 USA; Department of Orthopedic Surgery, University of California, 200 West Arbor Drive, San Diego, CA 92103 USA; Department of Radiology, Rady Children’s Hospital, 3030 Children’s Way, Suite 410, San Diego, CA 92123 USA

**Keywords:** Nonossifying fibromas, Distal tibia interosseous membrane, Etiology

## Abstract

**Purpose:**

Nonossifying fibromas (NOFs) present in a characteristic pattern in the distal tibia. Their predilection to this region and etiology remain imprecisely defined.

**Methods:**

We performed a retrospective chart review of patients between January 2003 and March 2014 for distal tibial NOFs. We then reviewed radiographs (XRs), computed tomography (CT), and magnetic resonance imaging (MRI) for specific lesion characteristics.

**Results:**

We identified 48 distal tibia NOFs in 47 patients (31 male, 16 female; mean age 12.3 years, range 6.9–17.8). This was the second most common location in our population (30 % of NOFs), behind the distal femur (42 %). Thirty-four lesions had CT and nine had MRI. Thirty-one percent were diagnosed by pathologic fracture. Ninety-six percent of lesions were located characteristically in the distal lateral tibia by plain radiograph, in direct communication with the distal extent of the interosseous membrane on 33 of the 34 (97 %) lesions with CT available for review and all nine (100 %) with MRI. The remaining two lesions occurred directly posterior.

**Conclusions:**

The vast majority of distal tibial NOFs occur in a distinct anatomic location at the distal extent of the interosseous membrane, which may have etiologic implications.

**Level of evidence:**

IV (case series).

## Introduction

Originally described in detail in 1942 by Jaffe and Lichtenstein, nonossifying fibromas (NOFs) are the most common benign lesion of bone seen in children [1–4]. They possess a characteristic radiographic (XR) appearance of radiolucent, eccentric, cortically based lesions with an internally bubbly appearance and sclerotic margins. Histologic findings consist of fibroblastic cells in a storiform pattern. Their natural history is gradual ossification and resolution with skeletal maturity [3]. However, little remains known about the etiology of these lesions [5].

The purpose of the present study was to characterize a large cohort of patients with NOFs isolated to the distal tibia by XR, computed tomography (CT), and magnetic resonance imaging (MRI). We empirically noted a predilection of these lesions to a characteristic location in the distal lateral tibia and predicted that such a pattern may emerge in a large series. Our aim was to further describe this anatomy with advanced imaging.

## Materials and methods

Institutional review board approval was sought and obtained. A retrospective chart review was performed on patients seen at Rady Children’s Hospital in San Diego between January 2003 and March 2014. Charts were queried for ICD-9 codes consistent with NOF and/or CPT codes consistent with curettage/grafting of bone lesion. These were then reviewed in detail for those with a diagnosis of distal tibia NOF (either by radiographic or histologic findings). Patients who did not have imaging available for review were excluded. No other exclusion criteria were employed. Charts were reviewed for patient demographics. Study patients were grouped by those diagnosed incidentally and those diagnosed by pathologic fracture. In the absence of pathologic fracture, lesions were characterized as “incidental” regardless of the presence or absence of pain, as this was not reliably elucidated in our retrospective chart review.

Radiographs from the time of diagnosis were reviewed for the presence and location of NOF. Lesions of the distal tibia radiographically consistent with NOF were included regardless of size. CT or MRI scans had also been performed around the time of diagnosis to further evaluate the lesion. These were reviewed to further characterize the relationship with surrounding soft tissue structures.

## Results

**Fig. 1:**
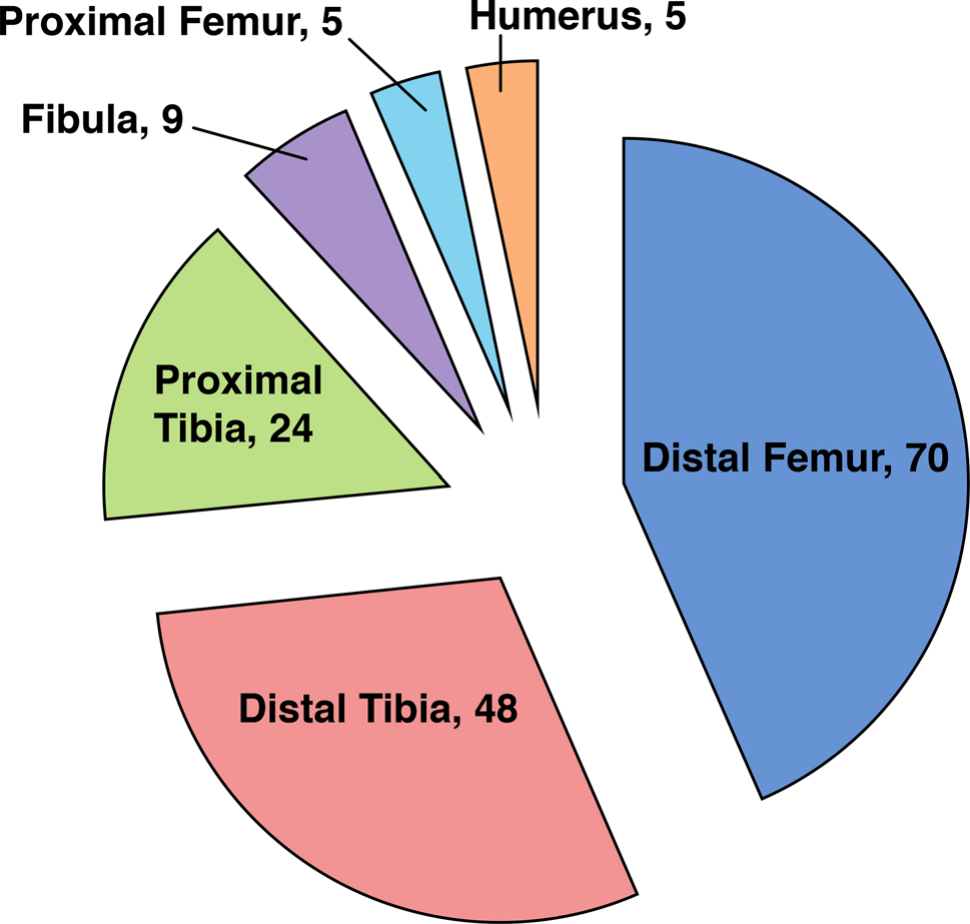
Distribution of nonossifying fibromas (NOFs) by location

**Fig. 2:**
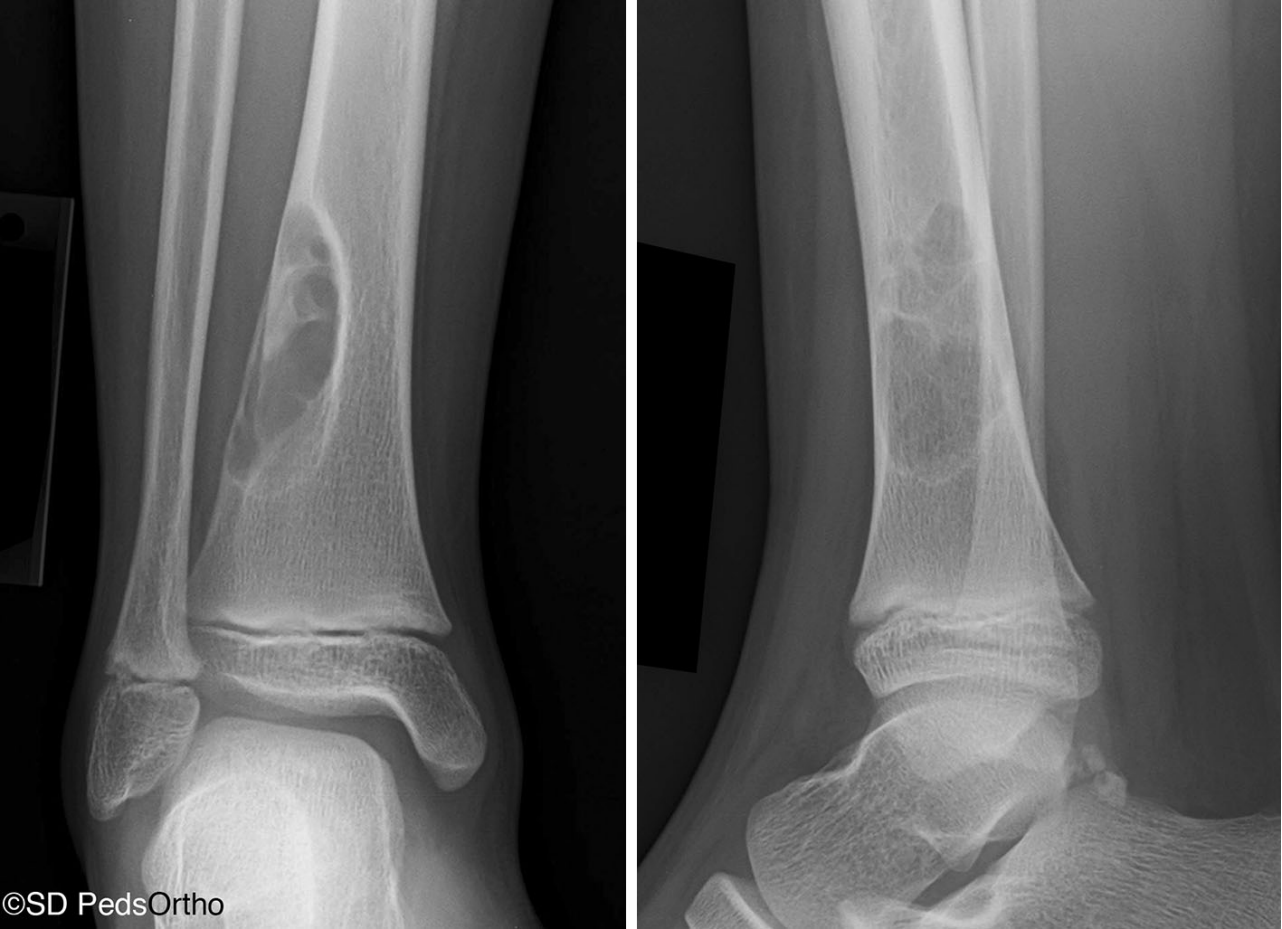
Characteristic location of a large NOF sprouting from the distal lateral tibia

Our query yielded 161 NOFs. Figure 1 demonstrates their anatomic distribution. Forty-eight lesions in 47 patients localized to the distal tibia. There was a male predilection (66 %) and the average age at initial XR was 12.3 years (range 6.9–17.8). Thirty-one presented with a pathologic fracture at the time of diagnosis. Radiographs were available for all patients, with 46 (96 %) lesions localizing to the distal and lateral aspect of the tibia, proximal to the physis (Fig. 2). The remaining two localized directly posteriorly at approximately the same height distally.

**Fig. 3:**
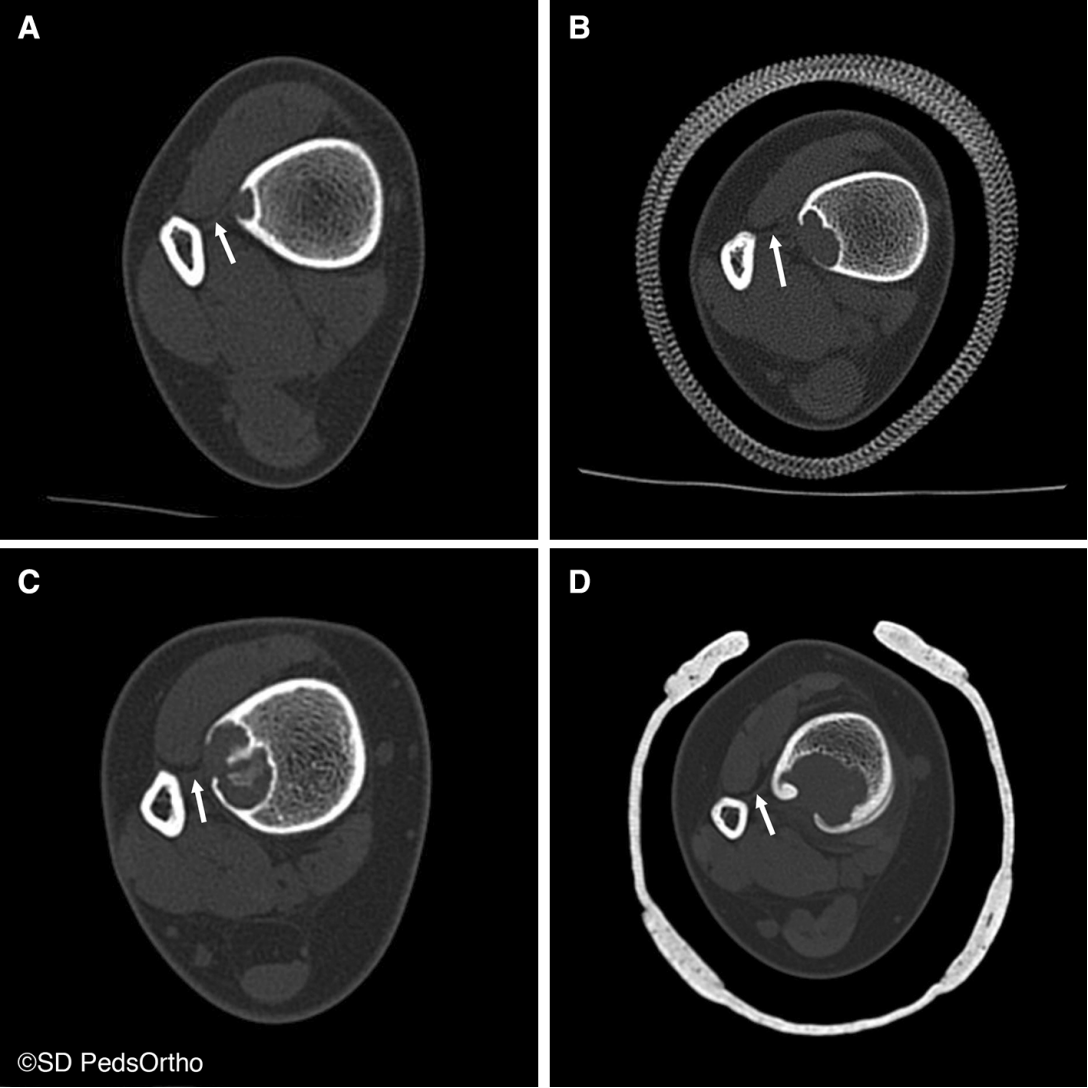
Progressively larger NOFs of the distal tibia in four different patients, revealing consistent lateral-to-medial growth originating adjacent to the interosseous membrane

**Fig. 4:**
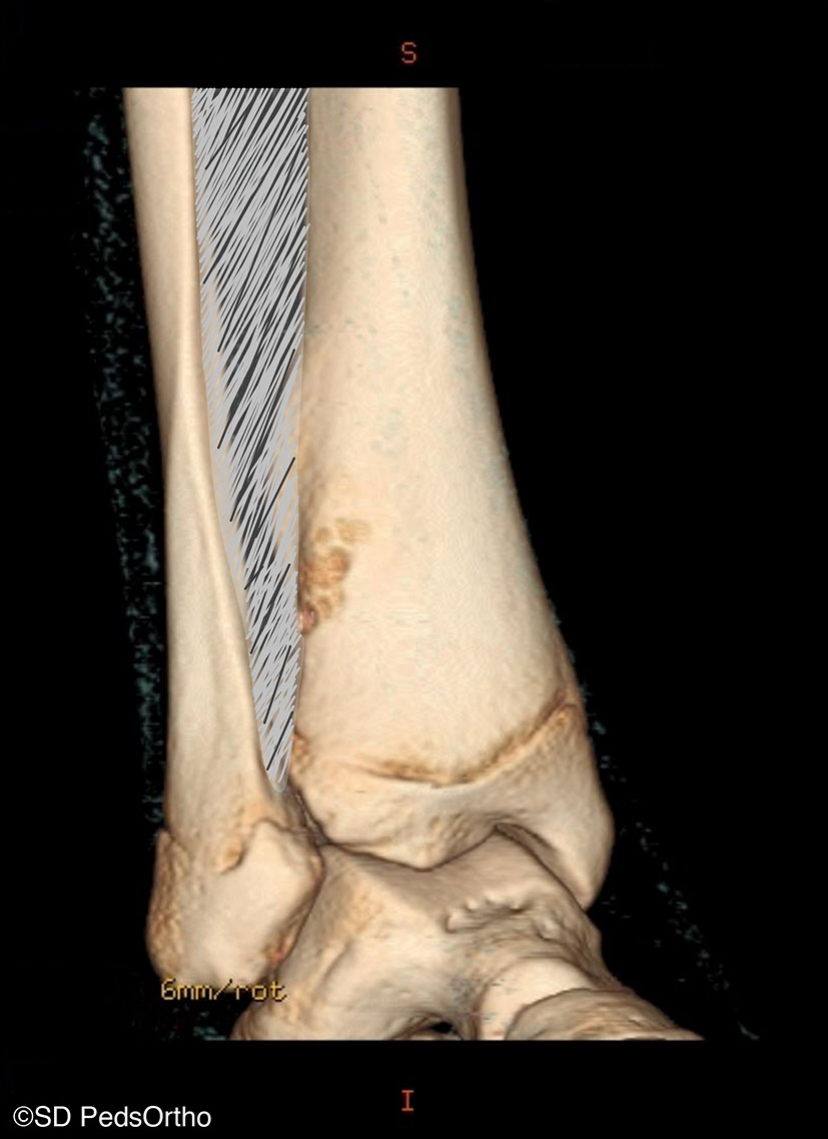
Three-dimensional reconstruction of computed tomography (CT) scan revealing an NOF with cortical breach located anterolaterally in the distal tibia

**Fig. 5:**
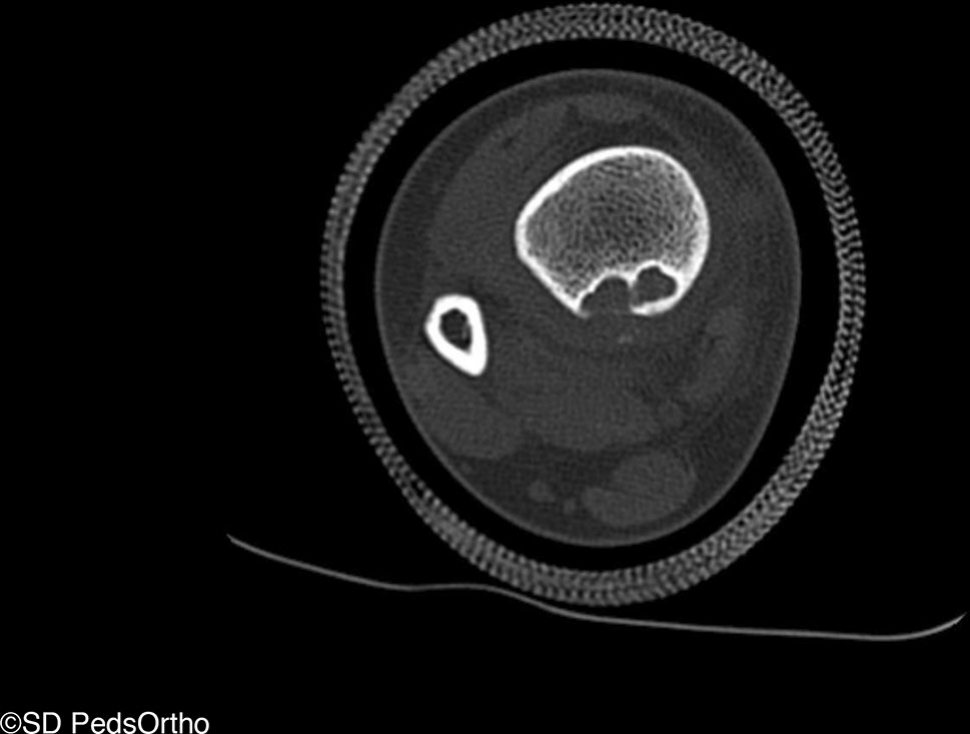
One of two lesions which localized directly posteriorly as opposed to adjacent to the interosseous membrane

**Fig. 6:**
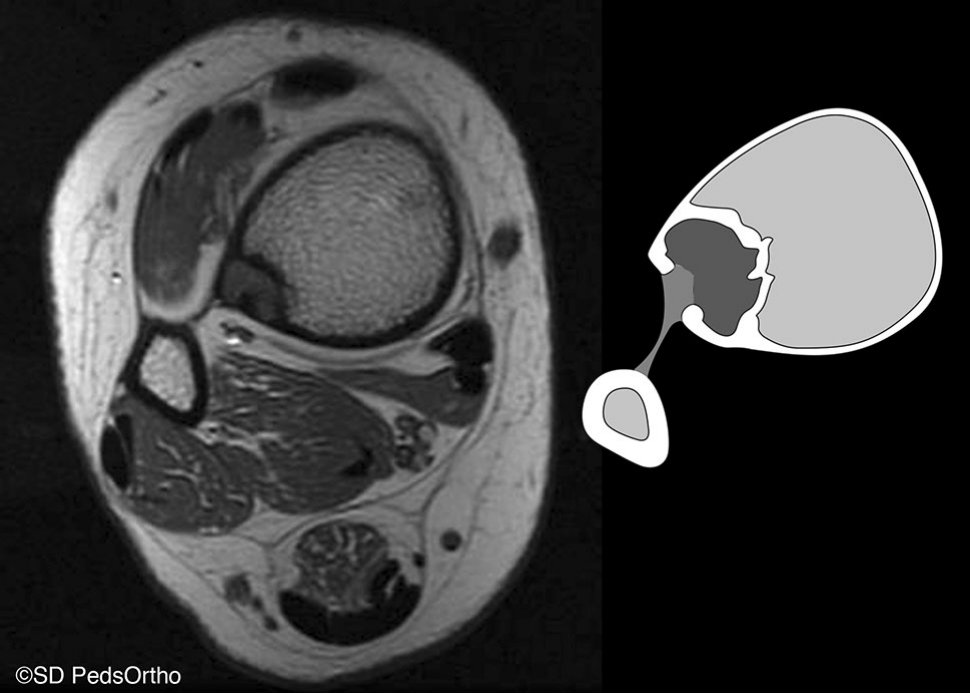
Magnetic resonance imaging (MRI) and schematic of continuity between the laterally localized NOF and the distal extent of the interosseous membrane

Thirty-four (71 %) patients had a CT scan available for review. Direct communication with the distal extent of the interosseous membrane was seen on 33 (97 %) lesions (Figs. 3 and 4), with the exception being the one of the two aforementioned posteriorly based lesions for which we had CT (Fig. 5). Cortical breach was noted in 28 scans. This breach localized to the interosseous membrane attachment on all except for the one posteriorly localized lesion. Nine (19 %) patients had an MRI available for review, and all showed continuity of the distal extent of the interosseous membrane with the distal, lateral extent of the NOF (Fig. 6).

## Discussion

The etiology of NOFs remains poorly understood, with predominant competing theories being that they arise either from bone marrow cell lineage or from a disturbance of the physis itself given their characteristic growth away from this structure [1, 5, 6]. The present study shows an eccentric localization of distal tibia lesions to the lateral metaphysis and a common relationship with the distal extent of the interosseous membrane on advanced imaging. The relationship between fibrous metaphyseal defects and the insertion of tendons and ligaments has been previously described by Ritschl et al. in 1988, though without the benefit of MRI confirmation [7]. While this association alone does not prove an etiologic relationship, we propose that the traction of the interosseous membrane could account for this localization in children. A similar process has been described in other lesions, such as distal femoral cortical irregularities localizing to the medial gastrocnemius origin on MRI, theoretically resulting from traction here during the relatively rapid growth of the posteromedial region of the distal femoral physis [8, 9].

**Fig. 7:**
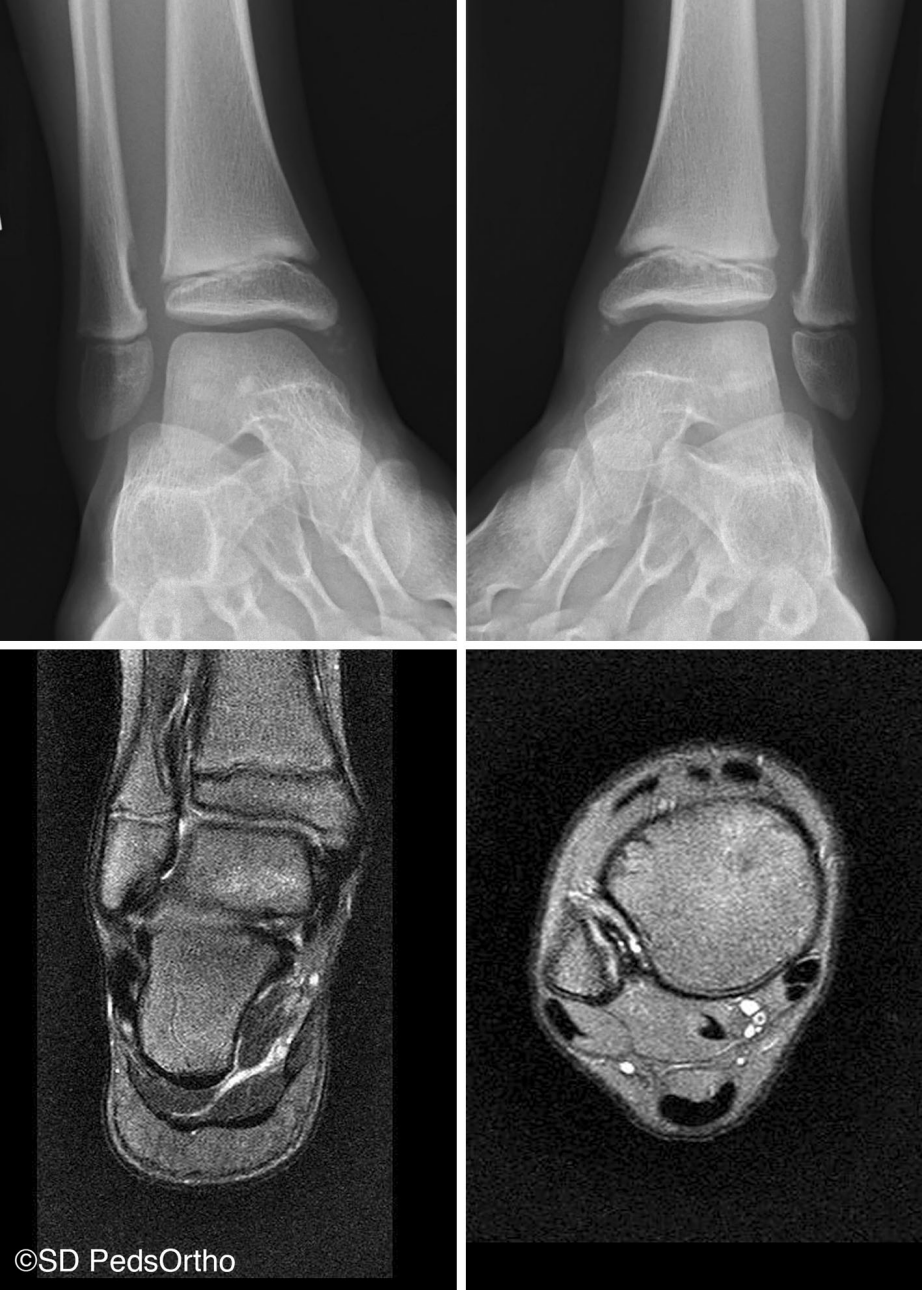
Bilateral distal fibular NOFs, each in communication with the distal extent of the interosseous ligament on MRI

Furthermore, there is a normal and well-described distal migration of the fibula relative to the tibia with growth of the pediatric ankle. This differential distal migration of the fibula to the tibia does not occur in children with a tibiofibular synostosis [10]. Such differential growth rates may provide traction on the interosseous ligament from fibular migration, contributing to NOF development. Alternatively, either longitudinal “pistoning” or the known external rotation of the fibula with respect to the tibia during normal gait may generate such traction. Interestingly, while not the focus of the current study, we have also encountered NOFs of the distal fibula, which, on MRI, can communicate directly with the distal extent of the interosseous membrane (Fig. 7), suggesting that such a process may affect either end of this structure. The two posteriorly localized lesions clearly show no relationship to the interosseous membrane (Fig. 5). Unfortunately, no MRI was available for review in either case, so precise soft tissue attachments could not be defined. One could theorize that an alternative structure (such as the posterior inferior tibiofibular ligament) may explain these variants.

The strength of the present series is in its inclusion of a large number of NOFs and a precise characterization of these lesions with advanced imaging. Nearly all NOFs were in a discrete location in the distal tibia and the size varied from 1 cm to very large, over 6 cm. We confirmed a male predilection of 2:1, as has been previously reported, but are unaware of a pathophysiologic explanation for such an observation [3, 5]. The retrospective nature of this series assumes all the limitations inherent to such a design. However, a wide spectrum of lesions were identified, from very small to very large (Fig. 3), and we, therefore, believe that a relatively representative spectrum of the disease was captured. Additionally, retrospective study in this case is unlikely to have introduced any bias with regards to the anatomic description of these lesions. In conclusion, the eccentric lateral localization of distal tibia NOFs and their anatomic relationship with the distal extent of the interosseous membrane may provide clues to the as yet undefined etiology of these lesions.

## References

[1] Jaffe HL, Lichtenstein L (1942). Non-osteogenic fibroma of bone. Am J Pathol.

[2] Caffey J (1955). On fibrous defects in cortical walls of growing tubular bones: their radiologic appearance, structure, prevalence, natural course, and diagnostic significance. Adv Pediatr.

[3] Betsy M, Kupersmith LM, Springfield DS (2004). Metaphyseal fibrous defects. J Am Acad Orthop Surg.

[4] De Mattos CBF, Binitie O, Dormans JP (2012). Pathological fractures in children. Bone Joint Res.

[5] Hatcher CH (1945). The pathogenesis of localized fibrous lesions in the metaphyses of long bones. Ann Surg.

[6] Cunningham JB, Ackerman LV (1956). Metaphyseal fibrous defects. J Bone Joint Surg Am.

[7] Ritschl P, Karnel F, Hajek P (1988). Fibrous metaphyseal defects—determination of their origin and natural history using a radiomorphological study. Skeletal Radiol.

[8] Suh JS, Cho JH, Shin KH (1996). MR appearance of distal femoral cortical irregularity (cortical desmoid). J Comput Assist Tomogr.

[9] Vieira RL, Bencardino JT, Rosenberg ZS (2011). MRI features of cortical desmoid in acute knee trauma. AJR Am J Roentgenol.

[10] Frick SL, Shoemaker S, Mubarak SJ (2001). Altered fibular growth patterns after tibiofibular synostosis in children. J Bone Joint Surg Am.

